# Systematic vs. Narrative Reviews in Sport and Exercise Psychology: Is Either Approach Superior to the Other?

**DOI:** 10.3389/fpsyg.2021.685082

**Published:** 2021-07-09

**Authors:** Philip Furley, Nadav Goldschmied

**Affiliations:** ^1^Institute for Training and Computer Science in Sport, German Sport University, Cologne, Germany; ^2^Department of Psychological Sciences, University of San Diego, San Diego, CA, United States

**Keywords:** secondary literature, PRISMA, Cochrane, literature review, critical review

## Introduction

With the explosion of published primary research there has been a parallel increase of secondary literature. Literature reviews substantially reduce the burden of readership, as readers are not only given a shortcut to the most important citations without having to review the wider literature pool on a certain topic but are also given an intelligent synthesis from the perspective of an expert. Although there are many published methodological guidelines (e.g., Grant and Booth, 2009; Higgins et al., 2019; Snyder, 2019) on how to conduct literature reviews, the interdisciplinary nature and broad scope of topics that are covered by the umbrella term of sport psychology calls for a critical discussion of how to adopt methodological guidelines developed in other fields. This paper addresses the increasing opinion (Mulrow et al., 1988; Higgins et al., 2019) that systematic reviews are the state of the art and superior to other forms of reviews as they have explicit and reproducible methodology and are assumed to be less biased than other reviews (Faggion et al., 2017).

We have made the experience and heard increasing reports in which editors and reviewers reject articles on the simple grounds of being “not systematic.” It is arguably more difficult and arduous to evaluate narrative reviews compared to systematic reviews given the precise methodological guidelines available for systematic reviews. However, this does not mean that systematic reviews are always the superior form of literature review, particularly in a diverse, interdisciplinary research field like sport and exercise psychology. Similar to Abraham Maslow's 1966 law of instrument (… “*it is tempting, if the only tool you have is a hammer, to treat everything as if it were a nail*.” p. 15) we will argue that different forms of literature review have different goals, are better suited for different questions or bodies of literature, cannot be ranked hierarchically, should be viewed as complementary, and need to be reviewed and evaluated differently.

## Systematic Review

In 1993 the Cochrane collaboration was founded with the goal to enhance healthcare knowledge and to facilitate health-related decision making by providing high-quality information by publishing and updating guidelines of how to synthesize knowledge in the form of systematic reviews (Higgins et al., 2019; Johnson and Hennessy, 2019; Alexander, 2020). The defining features of systematic reviews is a predetermined structured method to search, screen, select, appraise, and summarize primary research findings to answer a highly specific research question. The recommendation is to use an algorithmic approach by first explicitly stating the narrow research question and the exhaustive literature search methodology with precise predetermined inclusion and exclusion criteria. Usually a large body of articles are initially identified and subsequently whittled down to a lower number before the researcher starts to read the articles. The next step typically entails the creation of tables by extracting data from the primary research articles and mathematically summing the findings. The recommended approach for reporting the findings has been published on the Preferred Reporting Items for Systematic Reviews and Meta-Analyses (PRISMA) website (http://www.prisma-statement.org/) which originated from a report by Cynthia Mulrow and coauthors (Mulrow et al., 1988) that identified major pitfalls of published reviews (see AMSTAR for an alternative approach, Shea et al., 2017).

## Narrative Review

A narrative review can be defined as a scholarly report of a body of literature that includes interpretation and critique (Baumeister and Leary, 1997; MacLure, 2005; Grant and Booth, 2009; Greenhalgh et al., 2018). Although there are different types of narrative reviews that differ in their goals and approaches, it is important to note that none of these approaches is unsystematic, or *ad-hoc*, or even careless. A common goal of narrative reviews is to provide authoritative argument based on published primary evidence that is convincing to readers. Further goals are to enhance understanding of a topic and theory development (e.g., Dixon-Woods et al., 2006). A narrative review may or may not use systematic search methods with fixed inclusion/exclusion criteria. Narrative reviews are also often referred to as integrative (Torraco, 2005; Whittemore and Knafl, 2005) or critical review (Grant and Booth, 2009; Saunders and Rojon, 2011). There are many labels for different types of narrative reviews which we do not want to discuss in this commentary (MacLure, 2005; Grant and Booth, 2009; Greenhalgh et al., 2018), however they all share the distinctive goal of advancing understanding and theory development of a certain topic.

## Systematic or Narrative Review in Sport and Exercise Psychology?

It clearly depends on the aim of the researcher and the questions attempted to address that determine which type of approach to choose. However, people should not conflate “systematic” with superior quality and “narrative” with inferior quality (Greenhalgh et al., 2018):

This implicit evidence hierarchy (or pyramid) elevates the mechanistic processes of exhaustive search, wide exclusion and mathematical averaging over the thoughtful, in-depth, critically reflective processes of engagement with ideas (p. 3).

We do not consider it sensible to primarily educate students and young scholars in the technical skills of searching, sorting, utilizing inclusion/exclusion criteria, extracting data, and calculating summarizing statistics at the expense of understanding, argumentation, weighing evidence, writing, and formulating convincing narratives. Even though people have great confidence in numbers, which seems to be deeply rooted in human psychology (Porter, 2020), “*numbers or data have no way of speaking for themselves*” (Silver, 2012, p. 9). It is very unlikely that we will draw important conclusions and enhance cumulative understanding by simply extracting numbers from primary research without corresponding interpretation and theory, or as stated in the editorial of Psychological Bulletin: “*evidence synthesis that focuses purely on fancy statistical operations is unlikely to ‘tell the story' of a phenomenon*” (Johnson, 2021, p. 1).

The medical field has also acknowledged the problems of the mass production of systematic reviews. Prominent scholar Ioannidis (2016) critiqued the massive production of systematic reviews as this can be harmful due to the major prestige these types of publication: “*these instruments often serve mostly as easily produced publishable units or marketing tools*” (p. 485). In sport psychology there seems to be a similar trend, although there is the added difficulty that the methodologies used in these fields are very diverse and therefore cannot be easily be integrated in systematic reviews. While some research in sport psychology is focused on testing interventions (e.g., to improve performance or increasing exercise participation) a vast amount of research is focused on understanding the complex nature of the psychology of sport performance and exercise behavior. As research on the latter does not have an agreed-upon gold standard methodology like double-blind-randomized-controlled-trials, this research often requires narrative literature reviews that are focused on advancing theoretical understanding (e.g., Strauss, 2002; Nieuwenhuys and Oudejans, 2012). Considering calls for a renewed focus on theory development in psychological science (Muthukrishna and Henrich, 2019; Berkman and Wilson, 2021; Eronen and Bringmann, 2021) we consider narrative reviews highly important in this endeavor in sport psychology.

A common argument for systematic reviews over narrative reviews is that systematic reviews are less biased due to its dispassionate, instrumental nature that apparently leaves no room for subjectivity. However, this “view from nowhere perspective” is neither realistic nor even desirable as different perspectives and discourse are a prerequisite for scientific progress (Merton, 1938). Moreover, it is even questionable if the systematic review technique is superior in reducing bias given recent demonstrations that data have no way of speaking for themselves (Silberzahn et al., 2018).

Narrative reviews are also not without problems and can be performed badly. Authors might “cherry pick” certain primary research to bolster a certain opinion. However, the quality of a literature review should not be determined or influenced simply by the words “systematic” or “narrative” in the title. Although there are some distinguishing characteristics between the two broad categories “systematic” and “narrative” there are no clear boundaries between these categories and there are many subtypes that share features of both types of review categories. Therefore, it can be sensible to combine the “best of both worlds” by integrating systematic methodology and narrative approaches, which is precisely what characterizes good reviews (Johnson, 2021). Several recent publications (Johnson and Hennessy, 2019; Alexander, 2020; Hulland and Houston, 2020) have proposed guidelines of improving systematic reviews, especially regarding their role in theory development. However, these guidelines typically pertain to systematic reviews and not to narrative reviews and often implicitly demean narrative reviews by using terminology like “meandering narrative stroll” (Hulland and Houston, 2020, p. 352) or “simple narrative description” (Hulland and Houston, 2020, p. 353). Hence, the following section summarizes ways of improving narrative reviews and defending their role in the scholarship of sport psychology.

## Writing and Evaluating a Narrative Review

This section is not supposed to be a “How to Guide” of writing what we consider to be a good literature review. Instead we propose some guiding questions that might help authors give readers an interpretative overview of a topic with the aim of clearly highlighting the state of knowledge in a field, outlining how this knowledge was obtained, highlighting uncertainty in a field, and thereby pointing to the most important unanswered questions. No matter what type of review an author adopts they need to specify the topic and the goal of the review. Baumeister and Leary (1997) propose five goals of narrative reviews: (1) theory development; (2) theory evaluation; (3) survey the state of knowledge on a particular topic; (4) problem identification; (5) historical account of a particular research topic. Once the goal of a narrative review has been stated the following guiding questions (adapted from Baumeister and Leary, 1997; Saunders and Rojon, 2011) might be helpful for writing and evaluating the literature review. (1) Are the goals of the literature review explicitly stated and contextualized?; (2) Has the most relevant research on the topic been identified and included?; (3) Has the strength of evidence been addressed? (4) How consistent is the evidence and what factors are associated with variability? (5) Has the included primary research been critically evaluated and reported with a clear structure that is logical to the reader?; (6) Is primary research included that both supports and opposes the main arguments of the author? (7) Are explicit and comprehensible reasons mentioned for excluding primary literature; (8) Is empirical evidence clearly distinguished from personal or expert opinion?; (9) Are the arguments made logically and are they justified with valid evidence?; (10) Does the report point out areas of agreement and disagreement? (11) Are the conclusions straight-forward and logical?; (12) Does the report explicitly identify limitations and future research directions that follow from the review in a logical manner?

## Conclusion

The unreflective adoption of the systematic review approach that originated in the field of medicine as the gold standard of literature reviews has received hefty critique (Greenhalgh, 2012; Ioannidis, 2016; Greenhalgh et al., 2018). The problem of adopting the systematic review approach as gold standard in other fields has been vividly captured in the field of education by a report with the memorable title “Clarity bordering on stupidity:” where's the quality in systematic review? (MacLure, 2005). We do not question the merit of systematic reviews for some questions in the field of sport psychology but consider narrative reviews as equally important complementary approaches. Or stated in the words of Maslow (1966), just as carpenters need different tools to get different jobs done, (sport)psychological researchers need different tools for summarizing the quickly growing body of primary research. There is no hierarchy in these different approaches and the merit of each of these approaches needs to be evaluated differently. Hence, we neither recommend rejecting literature reviews on the simple grounds that they are “not systematic” nor do we recommend the simple rule of thumb to revise narrative reviews to be transformed to systematic reviews as “*the narrative review is not a poor cousin of the systematic review but a different and potentially complementary form of scholarship*” (Greenhalgh et al., 2018, p. 4).

## Author Contributions

PF wrote the first draft of the manuscript. NG contributed to this draft and provided critical feedback. All authors contributed to the article and approved the submitted version.

## Conflict of Interest

The authors declare that the research was conducted in the absence of any commercial or financial relationships that could be construed as a potential conflict of interest.
